# Motor atypicalities in infancy are associated with general developmental level at 2 years, but not autistic symptoms

**DOI:** 10.1177/1362361320918745

**Published:** 2020-05-15

**Authors:** Sheila Achermann, Pär Nyström, Sven Bölte, Terje Falck-Ytter

**Affiliations:** 1 Uppsala University, Sweden; 2 Karolinska Institutet, Sweden; 3 Stockholm County Council, Sweden; 4 Curtin University, Australia; 5 Swedish Collegium for Advanced Study (SCAS), Sweden

**Keywords:** autism spectrum disorder, infancy, interceptive action skills, motion capture technology, motor development

## Abstract

Atypical motor development has frequently been reported in infants at elevated likelihood for autism spectrum disorder. However, no previous study has used detailed motion capture technology to compare infant siblings of children with autism spectrum disorder and infant siblings with no familial history of autism spectrum disorder. We investigated reaching movements during an interceptive action task in 10-month-old infants using kinematic data with high spatiotemporal resolution. The results indicated that several measures were different in infants at elevated likelihood. However, longitudinal analyses revealed that while specific infant motor measures (e.g. number of movement units) were related to broad measures of general developmental level in toddlerhood, the associations with later autism spectrum disorder symptomatology were not significant. These findings confirm that some aspects of motor functioning are atypical in infants at elevated likelihood for autism spectrum disorder, but provide no support for the view that these issues are specifically linked to autism spectrum disorder symptoms, but may rather reflect neurodevelopment more generally.

**Lay abstract:**

Atypicalities in motor functioning are often observed in later born infant siblings of children with autism spectrum disorder. The goal of our study was to investigate motor functioning in infants with and without familial history of autism spectrum disorder. Specifically, we investigated how infants catch a ball that is rolling toward them following a non-straight path, a task that requires both efficient planning and execution. Their performance was measured using detailed three-dimensional motion capture technology. We found that several early motor functioning measures were different in infants with an older autistic sibling compared to controls. However, these early motor measures were not related to autistic symptoms at the age of 2 years. Instead, we found that some of the early motor measures were related to their subsequent non-social, general development. The findings of our study help us understand motor functioning early in life and how motor functioning is related to other aspects of development.

Autism spectrum disorder (ASD) is a neurodevelopmental condition characterized by atypicalities in various aspects of motor functioning (Fournier et al., 2010; Green et al., 2009; Leonard et al., 2014). While motor atypicalities are not included in the diagnostic criteria of ASD (American Psychiatric Association 2013), they are considered in the clinical practice and described as an associated feature of the condition in the *Diagnostic and Statistical Manual−fifth edition* (DSM-5). Motor functioning is closely intertwined with social (Gredeback & Daum, 2015; Gredeback & Falck-Ytter, 2015) and communicational skills (Mottonen et al., 2005; Willems & Hagoort, 2007), as well as variety of cognitive skills (Gerson & Woodward, 2014; Gottwald et al., 2016; Libertus et al., 2016). Therefore, it could be argued that motor functioning is not easily disentangled from the core symptomatology of ASD and that all ASD-defining behavioral domains need to be understood and studied in concert with motor functioning. In this study, we examined motor functioning on a microstructural level in infant with an older autistic sibling and infants with no familial history of ASD using an interceptive action task (i.e. reaching for and catching a moving target). In addition, we investigated whether early motor functioning was related to ASD symptoms and other developmental outcomes at 24 months of age.

## Early motor development in ASD

Earlier retrospective home video studies have suggested early motor atypicalities in sitting, crawling, and walking, as well as a lack of symmetry in movements and postures in infants later diagnosed with ASD (Esposito & Venuti, 2009; Teitelbaum et al., 1998). Today, early signs of ASD are mostly investigated by following infant siblings of autistic children over time, as the likelihood of a diagnosis is elevated in families with one child already on the spectrum (Constantino et al., 2010; Ozonoff et al., 2011; Sandin et al., 2014). Specifically, around 20% of infant siblings with at least one older autistic sibling go on to receive the diagnosis themselves (Messinger et al., 2015; Ozonoff et al., 2011; Sandin et al., 2014). Therefore, prospective longitudinal studies of infant siblings at elevated likelihood (EL) for ASD present a promising approach to detect early markers of ASD.

Although studies do not present an entirely homogeneous picture, overall it seems that motor atypicalities are associated with ASD from an early age (Bhat et al., 2011; Fournier et al., 2010; Jones et al., 2014; Leonard et al., 2014). However, it is important to underline that not all studies find clear differences between children with and without familial history of ASD (Iverson & Wozniak, 2007; Landa & Garrett-Mayer, 2006; Taffoni et al., 2019). In addition, studies have examined vastly different types of movements or motor assessments scales, requiring readers to interpret findings with nuance and caution.

Flanagan et al. (2012) observed a head lag during the pull-to-sit task in 6-month-old infant siblings of autistic children, an ability assumed to be developed by the age of 4 months (Bly, 1994). Along the same lines, Leezenbaum and Iverson (2019) found a slower development of advanced postures when analyzing postural trajectories of infant siblings of autistic children. Furthermore, infant siblings who later developed ASD showed lower motor activity levels in the first year of life (Zwaigenbaum et al., 2005) compared with infants with no familial history of ASD. However, these measures were based on parent report, which may not be a sufficiently precise and valid measure to capture subtle differences and may be confounded with other behavioral attributes.

When it comes to reaching movements, Focaroli et al. (2016) found slower reaches during a block task in children with an older autistic sibling compared to children with a neurotypical sibling. In contrast, Taffoni et al. (2019) observed no differences between infants with and without familial history of ASD in motor performance when infants reached for blocks and fit them into boxes. The absence of group differences may be due to the task as the level of difficulty varied largely in terms of number of insertion possibilities and starting orientations of the blocks. In addition, methodological challenges such as a rather small sample and large variability within both groups may underlie the absence of differences. Sacrey et al. (2018) examined qualitative aspects of reach-to-grasp movements in infancy. The results indicated that children with later ASD showed worse total scores on the qualitative Skilled Reaching Rating Scale (Sacrey et al., 2012). Movements of infants with later ASD were less well coordinated, according to this study. This finding adds to the literature on early motor differences and highlights the importance of qualitative components of movements.

Regarding performance on standardized tests, Iverson and Wozniak (2007) found no support for statistically significant group differences between infant with and without familial history of ASD in the average onset ages for several motor milestones. In addition, Landa and Garrett-Mayer (2006) found no significant group differences when using standardized measures of motor development at 6 months of age, but both gross and fine motor delays were reported in 14-month-old infants later diagnosed with ASD.

## Early motor measures and later outcome in development

Diminished ability to plan ahead and prospectively control movements may have a profound impact on the development in infants at EL for ASD, not only in non-social domains, but also on social interaction and communication. Impaired motor functioning restricts the ways in which infants can interact with the physical and social environment and learn from their own actions (Adolph, 1997; Bushnell & Boudreau, 1993; Gibson, 1988). Furthermore, poor motor coordination have been associated with ASD (Ghaziuddin & Butler, 1998), which in turn may affect autistic children’s psychosocial environment in the form of not getting involved in social physical play on the playground (Bar-Haim & Bart, 2006; Smyth & Anderson, 2000).

Motor functioning is important for general development and provides infants with a variety of adaptive skills and learning opportunities, which also affects developmental change in other areas. Within the embodied cognition framework, bodily experiences are crucial for cognition and development is shaped by embodied processes. Impaired motor functioning may therefore inhibit the relationship between motor ability and cognitive development (Eigsti, 2013). Along the same lines, in a longitudinal study of toddlers at EL for ASD, Sutera et al. (2007) found that motor skills at 2 years of age predicted ASD outcomes at age 4 years and that daily living skills were associated with early motor functioning. Identifying early emerging motor atypicalities may therefore have important implications for the outcome of infants at EL for ASD and exhibit clinical relevance for early intervention. However, it has not yet been determined whether motor atypicalities are unique to early ASD or whether motor atypicalities may reflect a general risk indicator for developmental concern or cognitive impairment (Johnson et al., 2015; Ozonoff et al., 2008).

## Components of motor functioning

To investigate motor functioning with more detailed measures, we will now break down the generic construct into different components and examined *motor planning, spatiotemporal prediction, and motor execution*.

*Motor planning* has been described as “a process of converting a current state and a desired state into a sequence of motor commands” (Gowen & Hamilton, 2013), which means detecting the hand’s current position and the target position and turning this information into a chain of motor actions. Although motor planning often begins even before a movement has started, it is generally operationalized as a reaction time (latency to movement onset). Several studies on individuals with ASD have found atypicalities in movement preparation in this group, such as longer times to plan and prepare movements (Ekberg et al., 2016; Glazebrook et al., 2006, 2008; Rinehart et al., 2006a, 2001).

Furthermore, to be able to *predict* exactly where something (here: the catch) is going to happen in the near future is a crucial aspect of motor planning in general and interceptive tasks in particular. From early on, infants are required to plan ahead and adjust actions according to future goals (von Hofsten, 2004). In an interceptive action task, the trajectory of a target has to be predicted and taken into account during the reach in order to compensate for the processing lags of the nervous system and the time it takes to carry out the motor task (Kayed & Van der Meer, 2009; van der Meer et al., 1994; von Hofsten, 2014; von Hofsten et al., 1998). Evidence from head tracking and reaching studies suggests that 6-month-old infants with no familial history of ASD are already able to extrapolate an object’s motion into the future and to reach predictively for a moving target (van der Meer et al., 1994; von Hofsten et al., 1998). Although motor atypicalities in ASD are frequently reported (Fournier et al., 2010; Leonard et al., 2014), results on prospective motor control are somewhat inconsistent and it is yet to be determined whether they present a key impairment or not (Gowen & Hamilton, 2013; Mostofsky et al., 2004).

Finally, even after a reach has been initiated, accurate and efficient *execution* is needed to complete the task. An action can be divided into movement units (MUs), each consisting of an acceleration and a deceleration phase (von Hofsten, 1991). Reaching in adults typically consists of a large first MU, followed by small adjustments toward the end of the manual action (Jeannerod, 1988; Marteniuk et al., 1987). In infants, more MUs are needed before the target is reached, and the number of MUs decreases with maturation (von Hofsten, 1991; von Hofsten & Lindhagen, 1979), presumably reflecting improvements in planning, prospective control, and execution. In addition, the peak velocity of the first MU, as a measure of prospective motor control, has been shown to be related to high-order executive control in early life (Gottwald et al., 2016). Therefore, examining kinematic profiles provides new, fine-grained insight into how manual actions are performed on a microstructural level (Gottwald et al., 2016, 2017; Gottwald & Gredeback, 2015; Kahrs et al., 2013). However, in young infants at EL for ASD, there is currently little data on the fine-grained nature of motor functioning. Focaroli et al. (2016) found that 18- to 36-month-old toddlers at EL for ASD reached more slowly (i.e. reduced mean acceleration) than neurotypical toddlers in a block task (reaching for a block and throwing it into a tray/stack it on a target block to build a tower), indicating atypicalities in motor functioning. Focaroli et al. (2016) interpreted the finding as evidence of early delays in motor skills in children at EL for ASD. Although this study is relevant, it is only using static objects, which put fewer demands on motor planning processes (i.e. the measurements will capture overall motor movement performance rather than on-line motor control). This is especially true if average measures of the whole reach are used, as previous studies have shown that young infants typically use several MUs in a reach.

## Interceptive action tasks in children with ASD

An impaired ability to catch balls in school-aged children with ASD has been observed (Whyatt & Craig, 2012, 2013a, 2013b). Whyatt and Craig (2013a) interpreted that the difficulties in successfully completing interceptive action tasks may be due to impairments in motor planning, possibly grounded in diminished perception–action coupling. However, in infancy, the ability to catch balls may not be impaired. We have previously investigated motor functioning applying an interceptive action task with 10-month-old infant siblings of children with ASD and infant siblings with no familial history of ASD (Ekberg et al., 2016), some of which are included also in the current report (see “Methods”). Based on video observation, Ekberg et al. (2016) found that infants at EL for ASD initiated the movement later than the control group when reaching for a ball rolling down an inclined surface. This result was interpreted as a possible sign of atypicality in prospective motor control. However, no group differences movement duration or successfully catching the target were found. It can be speculated that early differences in motor functioning may build over time or when demands in the environment increase, which may lead to the observed impairments in previous studies (Whyatt and Craig 2012, 2013a). Here, we build on and extend the previous finding by Ekberg et al. (2016) using motion capture technology, which allows for more precise measurement of movement parameters than video coding to help clarifying the underlying structure of affected and unaffected motor sub-components.

## Aim

The aim of this study was to use three-dimensional (3D) motion capture technology to investigate kinematic variables in infants at EL for ASD during an interceptive action task, and to examine how these measures relate to later development. We used the same task as the cross-sectional study by Ekberg et al. (2016), but included motion capture technology to tap into the microstructure of motor functioning in infancy assessing aspects of *motor planning, prediction, and execution*, and followed the infants until 24 months of age. Specifically, we used velocity profile analysis in 10-month-old infants at EL and low likelihood (LL) for ASD to identify sub-components of the reaches. As the current sample is partially overlapping with the sample studied in Ekberg et al. (2016) (see “Methods”), we expected our results of our group comparisons to be in line with the earlier finding on the task, showing a later initiation in infants at EL for ASD. As reported in the previous literature, we conceptualized this measure as motor planning (Gowen & Hamilton, 2013). To more directly assess prediction, we investigated how far along the future trajectory of the target the reach was directed. We labeled this variable prospective aiming. Then, we examined MUs (von Hofsten, 1991; von Hofsten & Lindhagen, 1979) in the EL and the LL group as the number and the characteristics of MUs are thought to be sensitive to subtle differences in motor planning, control and execution (Gottwald et al., 2017; von Hofsten, 1991). Based on the previous literature, we expected differences in all these three domains in infants at EL for ASD.

In terms of the longitudinal analyses, we focused on two key domains: general developmental level and ASD symptomatology. We reasoned that if early motor differences are specifically linked to ASD, they should correlate with ASD symptoms, and this association should not be explained by general developmental delays.

## Methods

### Participants

A total of 58 ten-month-old infants were included in the final sample, consisting of 39 infants (20 females) in the EL group and 19 infants (nine females) in the LL group. The groups were initially larger, but exclusion criteria for motion capture analyses and technical failure made the measurement unreliable on a subset of infants and these recordings were therefore excluded (see “Data analysis and reduction”).

All participants were part of the ongoing, longitudinal Early Autism Sweden (EASE) study (www.smasyskon.se). EASE follows infant siblings of children with ASD and infant siblings without a familial history of ASD from 5 to 36 months of age. Infants who had at least one older autistic sibling formed the EL group. Older siblings’ diagnostic status was confirmed via a psychologist-led interview with parents and inspection of medical records. EL infants were recruited through clinical units, advertisements, and the project website. Infants without familial history of ASD (in first- or second-degree relatives) who had at least one older typically developing sibling formed the LL group. These infants were recruited from the lab’s database of families who had previously expressed interest in participating in research. The infants included in the study were all born at full term (>36 weeks). No infant had any confirmed or suspected medical problems (including visual and auditory impairments). Of the 45 infants included in the analysis by Ekberg et al. (2016), 21 (12 EL- and 9 LL-infants) were also included in our sample.

An experienced psychologist assessed the infants’ developmental level using the Mullen Scales of Early Learning (MSEL; Mullen, 1995) at 10 and 24 months of age. To assess autistic symptoms, the Autism Diagnostic Observation Schedule-2 (ADOS-2; Lord et al. (2012) was administered by a psychologist with research-level reliability on the measure. In addition, socioeconomic status was estimated by family income and parental education level (Table 1).

**Table 1.** Participant characteristics by group (EL and LL), final samples (mean/*SD*).

Measure	EL *n* = 39 (20 females)	LL *n* = 19 (nine females)	Pairwise comparison (*p* value^a^)
Age (days)	313.44 (15.59)	302.42 (10.87)	0.006
MSEL total score^b^ at 10 months	101.77 (12.70)	105.05 (11.45)	0.281
MSEL VR^c^	54.56 (10.42)	57.16 (8.45)	0.429
MSEL FM^d^	55.69 (7.88)	60.32 (9.08)	0.051
MSEL RL^e^	46.54 (9.93)	42.84 (10.26)	0.193
MSEL EL^f^	46.59 (8.20)	47.79 (13.65)	0.641
MSEL total score at 24 months	95.42 (14.31)	105.94 (15.47)	0.020
ADOS comparison scores^g^ at 24 months	3.40 (2.27)	2.63 (1.96)	0.211
SES^h^	7.08 (1.99)	7.60 (1.80)	0.264

EL: elevated likelihood; LL: low likelihood; *SD*: standard deviation; MSEL: Mullen Scales of Early Learning; ADOS: Autism Diagnostic Observation Schedule.

a Mann–Whitney *U* test.

b Mullen Scales of Early Learning, Early Learning Composite Score.

c Visual Reception Subscale.

d Fine Motor Subscale.

e Receptive Language Subscale.

f Expressive Language Subscale.

g ADOS scores presented as comparison score from 1 to 10, allowing to evaluate change across modules and quantifying ASD severity relatively independent from individual characteristics (Lord et al., 2012).

h Socioeconomic status calculated on the basis of parental education and income (equal weighting), expressed as a *z* score.

The study was approved by the Regional Ethical Board in Stockholm and was conducted in accordance with the standards specified in the 1964 Declaration of Helsinki. All parents provided written informed consent.

### Procedure

The experiment and setup was the same as the one reported in Ekberg et al. (2016), and was part of a broad assessment, where families spent about 5 h in the lab. Upon arrival, the infant was familiarized with the staff and surroundings. Approximately half an hour after arriving at the lab, the experimental task reported in this article was administrated, which took about 10 min. The experimental task was followed by several other assessments including developmental assessments, eye tracking sessions, play observation, and parent–child interaction (plus breaks as needed; Achermann et al., 2020).

**Figure 1.**
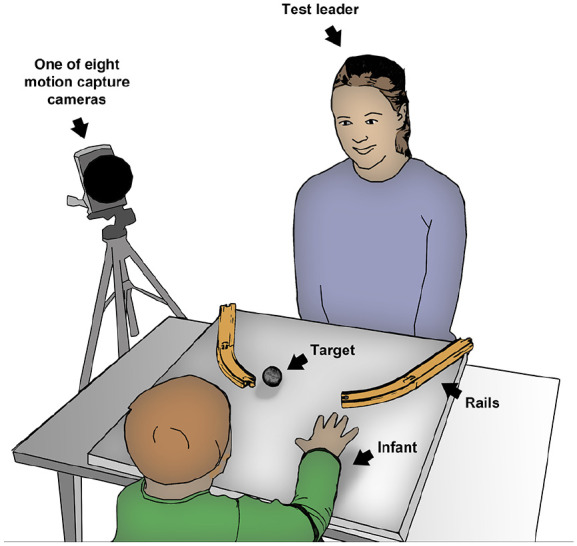
Sketch of the experimental setup with infant and test leader sitting opposite of each other at a quadratic table with adjustable tabletop.

The experimental task reported in this article was to catch a moving target, which was rolling toward the infant on a curvilinear path. During the task, the infant and the experimenter sat opposite of each other at a quadratic table (60 × 60 cm, Figure 1). The infant was seated on a high chair with the parent directly behind the child. On the tabletop were two toy rails serving as tracks to roll a ball from both the left and the right corner of the table. The tabletop was adjustable to two different degrees of inclination, resulting in two different rolling speeds. The lower inclination was at 2.8° from the horizontal plane, resulting in a rolling speed of approximately 25.09 cm/s (*SD* = 3.80) when rolling into the reaching space. The higher inclination was at 4.1° from the horizontal plane, resulting in a rolling speed of approximately 32.24 cm/s (*SD* = 4.19) when rolling into the reaching space. The reaching space was defined as the area where the ball was within approximate reach for the infant (325 mm from infant, see Ekberg et al., 2016).

After a short warm-up phase, which included a give and take interaction between the test leader and the infant to make the infant comfortable with the situation, the experiment started. The ball was introduced and, thereafter, trials started on the lower inclination of the tabletop with the lower rolling speed for the ball. At least four trials were completed, with two trials starting from the right corner and two trials starting from the left corner of the table, followed by an additional four trials at the higher inclination of the tabletop (Figure 1). The experimenter started the trial when the child’s attention was focused on the target. To avoid the impact of social motivation to complete the task, no social cues (e.g. vocalization, facial expression) were given before releasing the ball. If the test leader needed to capture the infant’s attention the ball was knocked against the table a few times to attract the infant’s attention. The infant had no restrictions in initiating the movement and was free to start to reach at any point. During a trial, the infant could watch the ball roll down the tracks for approximately 3 s before it left the tracks and entered into the reaching space. The end of each trial was marked by either the infant catching the ball or by the ball rolling off the tabletop. The reaching experiment took approximately 3 min to complete.

A motion tracking device (Qualisys Motion Capture Systems, Gothenburg, Sweden) was used for recording the targets’ and the infants’ movements. Data were recorded with an eight-camera 3D motion capture system at a sample rate of 240 Hz. Passive reflective markers (0.4 cm in diameter) were attached to the infants’ hands (i.e. the superficial branch between the index finger and the thumb) and head to track movements. The moving target in the task was a ball (4 cm in diameter) covered in a special retro-reflective tape designed for motion tracking. In addition, a video camera (Sony Handycam HD) was mounted above the table, filming the experiment from a bird’s-eye view.

### Data analysis and reduction

Analyses were based on video recordings and motion tracking data. The video analysis was completed with a frame-by-frame software (Mangold International INTERACT, Arnstorf, Germany), consistent with prior research by Ekberg et al. (2016). The video analysis was used to produce events for the motion tracking analysis. First, videos were coded for the beginning and the end of the reach, and in a next step, the outcome of the reach and the hand used for the reach (right, left, bimanual) was registered. Reaches were coded as either (1) *reach* (contact with the ball or within 2 cm) or (2) *other*. The category *other* was rather heterogeneous and included subcategories, such as experimenter errors, unsuccessful reaches, no reaching attempt at all, or non-task related actions in the reaching movement (e.g. hand flapping). The coding was naïve to group membership. To ensure high-quality data, analyses are based on *reaches*, while the category *other* was excluded. An interrater reliability analysis using Cohen’s Kappa was performed, as an additional rater double-coded 20% of the videos. The interrater reliability was high (Cohen’s kappa = 0.89, *p* < 0.001, 95% kappa confidence interval = 0.82–0.97). Subsequently, the information obtained from the video coding was used in the motion tracking analysis. Motion tracking data from the eight-camera 3D motion capture system (Qualisys) were used to extract kinematic variables of the reaching movement.

**Figure 2.**
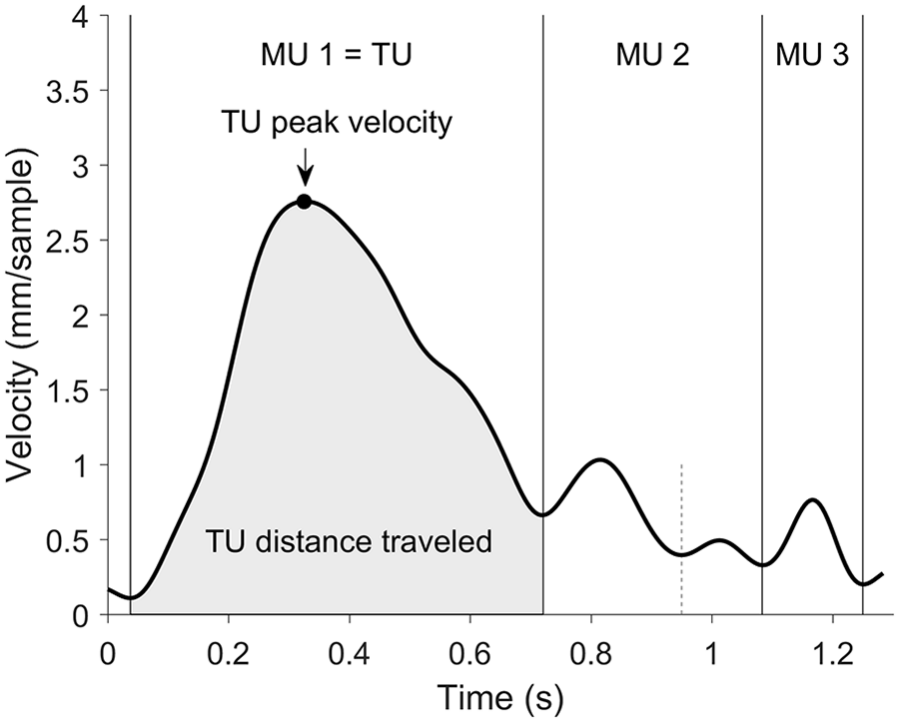
Example velocity profile from one of the participating infants. A reach is divided into movement units (MUs) from their local minima. The transport unit (TU) represents the largest MU toward the target. The main kinematic variables were the TU peak velocity (as shown by arrow) and TU distance traveled (gray area). The short dashed line indicates a local minimum where two MUs were merged into one.

The data was analyzed in MATLAB r2015b using the TimeStudio framework (http://timestudioproject.com, Nyström et al., 2016), an open source scientific workflow system. All analysis steps and algorithms described below are available using *uwid ts-095-8ae*, with sensitive participant information removed. As a first step and in line with prior research (Gottwald et al., 2017; Gronqvist et al., 2011), data were smoothened with a fourth-order Butterworth low-pass filter at 10 Hz. Next, 3D velocity was calculated and filtered using the same Butterworth filter to decrease measurement noise on the velocity profile. MUs were then extracted based on the interpolated velocity profile for each trial by taking every local minima as the onset of individual MUs (Figure 2). If the peak velocity of a MU was less than 8 mm/s above an adjacent local minimum, the MU was not considered to reflect an intended on-line adjustment of the reach, and the MU was merged into the adjacent MU. This merging threshold was based on blinded visual inspection of the data; a lower threshold resulted in a disproportionate amount of MUs, while a higher threshold filtered out potentially relevant on-line adjustments of the reach. This approach served as an artifact removal filter, similar to the merging strategies used in Gottwald et al. (2017).

Then, data were reduced because not all infants contributed with both motion tracking data and video recordings. A total of 118 infants completed the 10-month-visit; however, 35 infants were excluded due to missing data and technical problems, leaving 83 infants with both video recordings and motion tracking data that contributed to a total of 779 trials.

Finally, data analysis and reduction continued on trial level. After visual inspection, trials with less than 50% motion tracking data were excluded from the sample to ensure high-quality data (*n* = 260). Further exclusion criteria were as follows: if the hand moved less than 100 mm toward the target (*n* = 45, i.e. not enough movement), manual rejection (*n* = 3, that is, data contained noise artifacts), unknown if reach within 2 cm (*n* = 7, that is, technical problems), peak velocity higher than 100 mm/sample (*n* = 16, that is, data contained noise artifacts), straightness of the approach path higher than 5 (*n* = 7, that is, devious approach path due to hand flapping during reach), unknown hand (*n* = 2, that is, technical problems), number of MUs higher than 6 (*n* = 2, that is, data contained noise artifacts), and outlier values (*n* = 4, that is, data contained noise artifacts). At this point of analysis, only reaches that came at least 2 cm within the radius of the target withstood the exclusion criteria.

The final sample consisted of 58 infants that contributed with 419 trials in total. Infants included in the final sample completed at least three reaches. Similar to Gottwald et al. (2016, 2017), we examined the kinematic profiles of reaching movements on the level of the MU. Here, we investigated the transport unit (TU), defined as the MU that moved the hand the farthest toward the target.

### Statistical analysis

Linear Mixed Models were fitted using the MATLAB “fitlme” command and a Maximum Likelihood fitting method, from within the TimeStudio workflow. The model was specified as “dv ~ group + slope + (1|subject),” where dv was one of six dependent variables, group (HIGH/LOW), and slope (FLAT/STEEP) were fixed factors, and subject was treated as a random factor with random intercept (to account for differences in the number of trials between subjects). We initially included an interaction term between group and slope, but as there were no significant effects in any analyses, the interaction term was therefore removed. Yet, slope had a marginally significant main effect on the distance traveled during the TU (*p* = 0.092). Slope was therefore kept as a covariate, but is not further discussed.

The dependent variables included: (1) the ball’s position (in mm) at the beginning of the TU as an indication of *motor planning* (initiation), measured in distance to target (mm), (2) *prediction* of the reach, defined by the intersection of the ball’s and the hand’s directions during the TU (projected on the table surface), and using the time (in ms) for the ball to reach the intersection as the unit of prospective aiming (i.e. how much time ahead does the infants aim: positive values are prospective and indicate aiming in front of the ball, and negative values indicate aiming that lags behind the ball). Finally, MU *analysis* included kinematic variables: (3) peak velocity of the TU (in mm/sample), (4) distance traveled during the TU (in mm/sample), (5) the straightness of the approach path during the TU (obtained by the movement trajectory during the TU divided by the shortest distance between the beginning and the end of the TU, that is, values close to 1 indicate a straight approach, whereas high values imply a devious approach path (von Hofsten, 1979)), and (6) the number of MU as an indicator for the efficiency and straightness of the movement (von Hofsten, 1991; von Hofsten & Lindhagen, 1979).

To investigate potential longitudinal relations, we first calculated the Pearson correlations between the motor measures and 24-month ADOS-2 data. In the case of significant correlations, we then tested whether the association remained controlling for MSEL 24-month data (partial correlation). We also explored the zero-order correlations between the motor measures and the covariate (MSEL).

## Results

### General performance

On average, participating infants contributed seven reaches (EL *M* = 7.10 trials, *SD* = 2.37; LL *M* = 7.00 trials, *t*(56) = −0.15, *p* > 0.25). Regarding the coding of reaches (*reach* or *other*), no groups differences were found in the amount of reaches (within 2 cm) for a moving target (EL *M* = 82.94%, *SD* = 19.86; LL *M* = 81.60%, *SD* = 16.06; *t*(71) = −0.29, *p* > 0.25), replicating earlier data on equal performance between groups (Ekberg et al., 2016). We further examined whether there were differences in actually catching a ball or reaching within 2 cm, resulting in a ratio of catches to reaches. This measure was an attempt to characterize a biomechanical reaction. Kinematic measures in the main analyses are closely related and dependent on several other factors. The ratio of catches to reaches, however, provides us with a clean and direct connection with the motor outcome. Interestingly, no group differences were found when comparing the ratio of catches to reaches, as the participating infants (irrespective of ASD likelihood) were equally successful at catching a moving target (EL *M* = 34.88%, *SD* = 23.03; LL *M* = 28.15%, *SD* = 28.17, *t*(56) = −0.10, *p* > 0.25).

**Figure 3.**
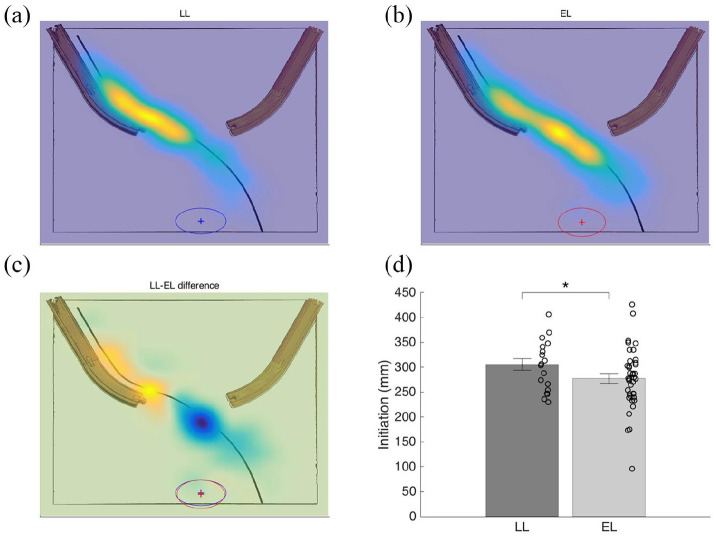
Motor planning (initiation) in LL and EL infants. Heat maps on the ball’s position on the trajectory relative to when the transport unit (TU) started and the hand position at TU start (mean denoted by +, whereas ellipses show standard deviation in *x* and *y* dimensions). (a) Normalized heat map displaying ball’s *X*- and *Y*-position on the trajectory when the TU started in the LL group. (b) Normalized heat map showing the ball’s position on the trajectory when the TU unit started in the EL group. (c) Normalized difference map highlighting the group differences on the trajectory relative to when the TU started. (d) Initiation as a function of group (measured in distance to target, mm, at the onset of the TU of the reach). *p < 0.05.

Differences in the starting position of the hand could confound subsequent kinematic data, but we found no differences between the EL and LL group in terms of the spatial coordinates of the hand at onset (*X*-position *F*(1, 393) = 0.22, *p* > 0.25; *Y*-position *F*(1, 393) = 0.04, *p* > 0.25; see Figure 3 for an illustration of these null results). In cases where infants missed or did not attempt to reach for the moving target, data could not be analyzed due to data quality restrictions and missing values.

### Main results

#### Motor planning (initiation)

As expected, and consistent with our earlier analysis on a subset of the current participants (Ekberg et al., 2016), at the time of the onset of the TU (the main unit of the reach), the ball was farther away in the LL group (*M* = 296.16 mm, *SE* = 5.53, 95% CI (285.29, 307.03)) than in the EL (*M* = 284.91 mm, *SE* = 5.50, 95% CI (263.23, 306.60)), *F*(1, 375) = 4.18, *p* = 0.042 (Figure 3).

**Figure 4.**
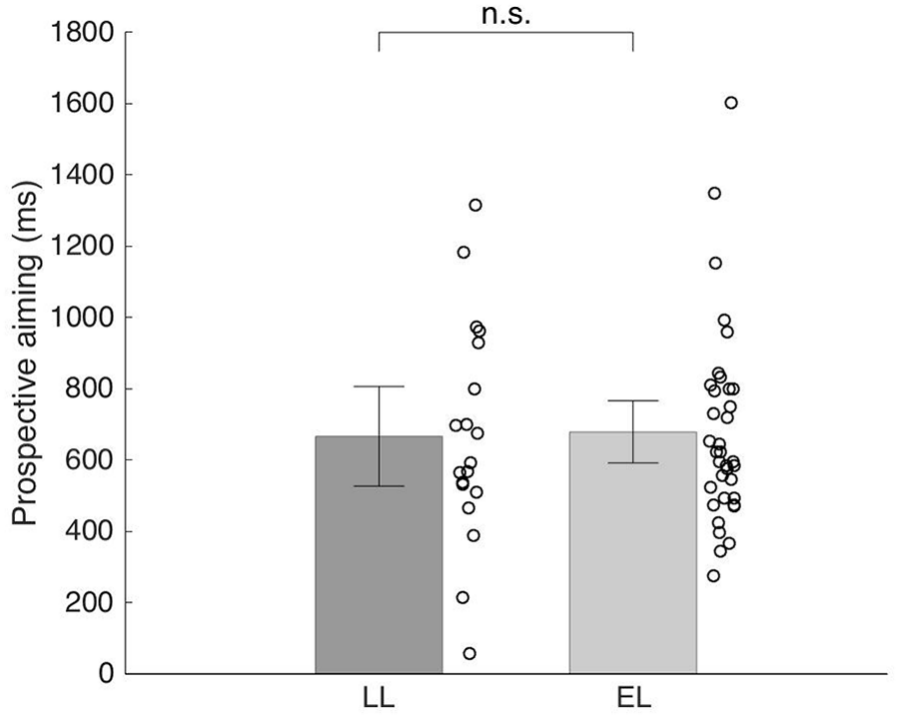
Prediction in LL and EL infants. Prospective aiming operationalized as how far along the future trajectory of the ball (expressed in ms) the reach is directed. Thus, a score of 1000 ms means that an infant directed his or her reach toward a position the ball would be in 1000 ms. Error bars represent standard errors. Circles represent averaged individual data points.

Prediction. Both LL and EL groups aimed prospectively toward the target and no group differences were detected (LL *M* = 648.79, *SE* = 36.86; EL *M* = 677.06, *SE* = 36.68; *F*(1, 224) = 0.59, *p* > 0.25, Figure 4).

#### Analysis of MUs

**Figure 5.**
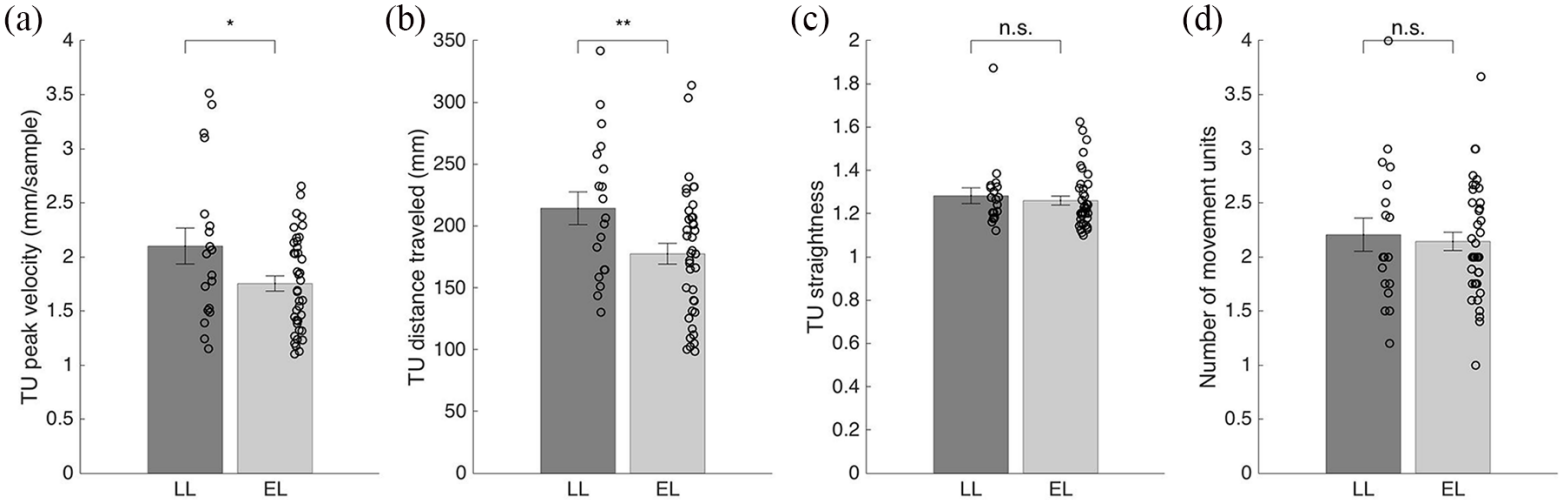
Analysis of movement units (MU). Illustration of main kinematic variables shown by averaged individual data points: (a) Transport unit (TU) peak velocity in mm/sample for the LL and the EL group. (b) TU distance traveled in mm for both groups. (c) Straightness of the TU for LL and EL group. (d) Number of MU during the reaching movement for LL and EL group. *p < 0.05. **p < 0.01 in main analyses. Error bars represent standard errors.

We found a group difference in the peak velocity of the TU and in its distance traveled. Peak velocity of the TU was significantly lower in the EL group (*M* = 1.77 mm/sample, *SE* = 0.07, 95% CI (1.48, 2.07)) than in the LL group (*M* = 1.94 mm/sample, *SE* = 0.08, 95% CI (1.80, 2.10)), *F*(1, 392) = 5.19, *p* = 0.023. Congruently, the distance traveled during the TU was significantly lower in the EL group (*M* = 177.89 mm, *SE* = 7.13, 95% CI (149.81, 205.97)) than in the LL group (*M* = 196.85 mm, *SE* = 7.15, 95% CI (182.79, 210.90)), *F*(1, 392) = 7.06, *p* = 0.008. However, the groups did not differ in the straightness of the TU (LL *M* = 1.27, *SE* = 0.02; EL *M* = 1.26, *SE* = 0.02; *F*(1, 392) = 0.23, *p* > 0.25) nor the number of MU during the reach (LL *M* = 2.18, *SE* = 0.06; EL *M* = 2.12, *SE* = 0.06; *F*(1, 407) = 1.00, *p* > 0.25, Figure 5).

#### Motor measures at 10 months in relation to later autistic symptoms and developmental level at 24 months

We found no indication that worse performance in terms of any of the motor measures at 10 months were related to more ASD symptoms (ADOS comparison score and subscales) at 24 months. This was true for the whole sample, and for the LL group separately. In the EL group, we found a trend toward a correlation between the peak velocity of the TU and later ADOS Comparison score (*r* = 0.314, *p* = 0.055); however, it is notable that the direction of this association was in the opposite direction as predicted.

**Figure 6.**
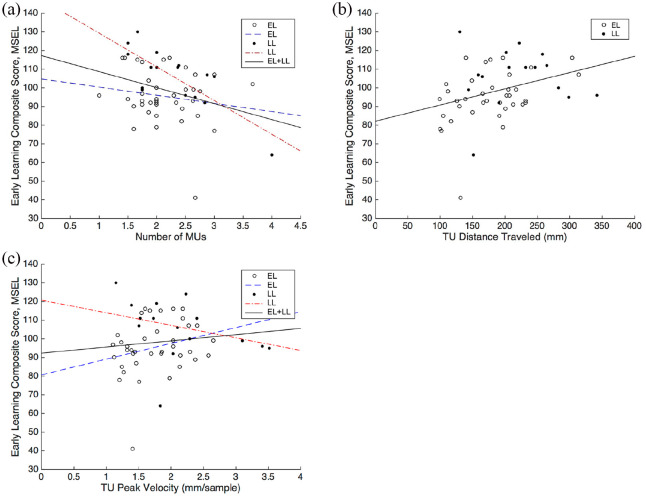
(a) Scatterplot of number of movement units (MU) against the Early Learning Composite Score on the MSEL with separate regression lines for each group (due to the significant interaction term; see main text for details). (b) Scatterplot of distance traveled (of the TU) against the Early Learning Composite Score on the MSEL with a regression line for the total sample. (c) Scatterplot of peak velocity (of the TU) against the Early Learning Composite Score on the MSEL with separate regression lines for each group. Full circles represent trials of LL infants, whereas open circles represent trials of EL infants.

A higher number of MUs during reaching at 10 months was associated with having a lower Early Learning Composite Score on the MSEL in the total sample (*r* = −.323, *p* = 0.017; Figure 6(a)). In addition, longer TUs (distance traveled) during reaching were associated with a higher Early Learning Composite Score on the MSEL in the total sample (*r* = 0.329, *p* = 0.015; Figure 6(b)).

To explore these correlations further, we conducted regression analyses to study the potential interaction between group and each motor measure. A regression analysis with group, number of MU and the interaction term (group × number of MUs) and the MSEL score as dependent variable, revealed a significant effect of group (*β* = 1.28, *t*(53) = −2.83, *p* = 0.007), number of MU (*β* = 0.68, *t*(53) = −3.56, *p* < 0.001) and an interaction effect (*β* = 0.97, *t*(53) = 2.11, *p* = 0.040; Figure 6(a)). For the peak velocity (TU), group (*β* = −1.21, *t*(53) = −2.66, *p* = 0.010) and the interaction term (group × peak velocity, *β* = 0.88, *t*(53) = 2.07, *p* = 0.044; Figure 6(c)) were significant predictors of the MSEL score at 24 months. For the remaining variables (distance traveled during the TU, straightness of the TU, prospective aiming and motor planning), the regression analyses did not show any significant interaction effects involving group.

In light of the significant interaction terms for number of MU and peak velocity of the TU, we analyzed these variables in each group separately. In the EL group, there was no significant association between the MSEL score at 24 months and neither of the two motor variables. In the LL group, a lower number of movements units was significantly correlated with a higher MSEL score (*r* = −.786, *p* < 0.001; Figure 6(a)), while the association between peak velocity (TU) and MSEL score at 24 months did not reach significance.

## Discussion

Our findings highlight some previously unrecognized and rather subtle differences between infants at elevated and LL for ASD in an interceptive action task. Some of these results, which were obtained using advanced motion capture technology, reveal differences not observable with traditional methods. Regarding motor planning when initiating a reach, the results are similar to earlier findings on the task, suggesting later initiation on average in infants with familial history of ASD (Ekberg et al. 2016, Figure 3). However, in terms of prospective aiming (Figure 4) we found no indication of group differences. Next, the analysis of MUs suggested group differences in motor execution and planning abilities, as the peak velocity of the TU and length of the TU (distance traveled) were on average lower in the EL group compared to the LL group (Figure 5). These findings are broadly in line with previous studies indicating differences in motor execution in infants and young children with later ASD (Focaroli et al., 2016; Rinehart et al., 2006b). Regarding the remaining kinematic variables, the straightness of the TU and the number of MUs, no indications of group differences (LL vs EL) were found. Taken together, our findings illustrate a complex pattern of differences, as well as similarities in early motor measures in infants at elevated and LL for ASD.

### Early motor measures and later outcomes

In addition to group comparisons of early motor measures at 10 months of age, we investigated the longitudinal associations between early motor measures and later autistic symptoms and developmental level at 24 months. Surprisingly, there was no significant association between early motor measures and ASD symptoms at 24 months. In fact, descriptively, for the peak velocity of the TU, there was a trend in the reverse direction than expected (higher velocity during reaching in infancy—less autism symptoms in toddlerhood).

Although not linked to ASD symptoms in our study, we found several significant correlations between early motor measures and broad measures of general developmental level at 24 months (MSEL) (Figure 6). Thus, the data in this report could indicate that early differences in motor functioning signal a more generalized delay in development. A longer TU (distance traveled) was associated with a higher Early Learning Composite Score on the MSEL and this relation was similar in EL and LL groups. Peak velocity (TU) was positively correlated with the later Early Learning Composite Score (MSEL). Furthermore, we found that a lower number of MUs during reaching was associated with a higher Early Learning Composite Score on the MSEL, although this association reached significance only in the LL group. For prospective aiming and for the straightness of the TU, we found no indications for longitudinal relationships. Regarding motor planning, we found a group difference (LL vs EL), but no longitudinal associations. This pattern is difficult to interpret and the lack of relationship to the MSEL or the ADOS-2 suggests that this measure may not be very informative clinically. Thus, our findings suggest a relationship between early motor measures and broad developmental level at later ages. Early motor differences may not only have consequences related to the motor domain, they may also be consequential for later development more generally (Bhat et al., 2012; Dziuk et al., 2007; Gottwald et al., 2016; Iverson et al., 2019; Sacrey et al., 2014). However, these early differences do not seem to have a relationship to ASD subsequent symptoms.

Taken together, our results tentatively suggest that there are certain motor measures—the number of MUs, the distance and the velocity of the TU—that may be particularly interesting from a developmental perspective and worth focusing on in future research. Importantly, differences between the EL and the LL group appeared for some measures but not for others, suggesting that it is critical to have sufficiently fine-grained methods to detect all underlying sub-domains of interest. These results are potentially interesting as they may hint toward differential developmental pathways in the two groups. While it is important to explore this hypothesis further in future work, we want to emphasize that this result was unexpected and should be interpreted with necessary caution. In addition, it is worth noting, that in the MU analysis, different components of motor functioning are closely related, such as a fast movement can cover a longer distance during the same amount of time and will inherently show higher velocity. Thus, we do not claim that our analysis of MU and its characteristics necessarily present independent results.

### Limitations

The study has some important limitations, with the lack of diagnostic outcome data at 36 months (endpoint in the EASE study) being an important one. The current sample was small, yielding a group based analysis of diagnostic status not feasible. However, it is important to stress that motor functioning, particularly in light of the results of this study, is relevant not only in the context of ASD. Furthermore, impairments in motor functioning are common in other neurodevelopmental disorders, such as in attention deficit hyperactivity disorder, motor coordination disorder, and intellectual disability. By following up the current sample to even later ages, we will be able to compare kinematic variables on interceptive action skills across a range of developmental outcomes. Another limitation is that, due to exclusion criteria regardless of group status, our analyses only included successful reaches. For future work, analyzing unsuccessful reaches (which in the case of infants include a wider variety of alternative actions, including doing nothing) may give further insight into potential differential motor patterns and other differences. A further limitation is that during a period of time in the beginning of the data collection, motion capture resulted in data loss due to an undetected technical problem. However, all motion capture data from the remaining infants (83 of the initial 118) underwent substantial quality checking, including visual inspection of individual trials, as well as various exclusion criteria to ensure that the final sample (*n* = 58) consisted of high quality data. While the technical problems were unfortunate and resulted in loss of statistical power, these issues are unlikely to explain the differences observed in this study between elevated and LL children as they were linked to a time period, not to the characteristics of the infants. Next, it is important to acknowledge that there is variability in the development of reaching skills and not all 10-month-olds are necessarily at the same developmental level. This variability is also reflected within each group on all of our motor variables. Importantly however, both groups had similar ratios of successful reaches.

Furthermore, our results do not tell whether the observed differences between EL and LL infants reflect delayed or atypical development, as we only examined motor variables at 10 months of age. Due to absent longitudinal data on motor measures, we cannot know whether the pattern displayed by the EL infants is similar to that seen in younger LL infants (i.e. delayed), or whether it is unlike anything seen in LL infants (i.e. atypical).

Finally, it is important to acknowledge that while our study represents a detailed analysis of a complex manual motor task, there may be aspects of motor control which are not well covered by this task and which indeed point toward a specific relationship to ASD (e.g. qualitative ratings of reaches and grasping and later ASD as suggested by Sacrey et al. (2018)).

## Conclusion

This study, which to the best of our knowledge is the first to use advanced motion capture technology in infants at EL for ASD, finds that early motor measures are associated with later developmental level, but not with ASD symptoms. These results contribute with novel insights to the field’s current understanding of the relationship between motor development and autistic symptoms, and may have implications to promote better assessments and possibly interventions for children at EL for ASD.
